# Prevalence and clinical characteristics of metabolically healthy obese versus metabolically unhealthy obese school children

**DOI:** 10.3389/fendo.2022.971202

**Published:** 2022-08-22

**Authors:** Ruziana Mona Wan Mohd Zin, Muhammad Yazid Jalaludin, Abqariyah Yahya, Ahmad Kamil Nur Zati Iwani, Fuziah Md Zain, Janet Yeow Hua Hong, Abdul Halim Mokhtar, Wan Nazaimoon Wan Mohamud

**Affiliations:** ^1^ Endocrine and Metabolic Unit, Institute for Medical Research, National Institutes of Health, Ministry of Health Malaysia, Setia Alam, Selangor, Malaysia; ^2^ Department of Paediatrics, Faculty of Medicine, Universiti Malaya, Kuala Lumpur, Malaysia; ^3^ Department of Social and Preventive Medicine, Faculty of Medicine, Universiti Malaya, Kuala Lumpur, Malaysia; ^4^ Department of Paediatrics, Hospital Putrajaya, Ministry of Health Malaysia, Putrajaya, Malaysia; ^5^ Department of Sports Medicine, Faculty of Medicine, Universiti Malaya, Kuala Lumpur, Malaysia

**Keywords:** obesity, children, metabolically healthy obese, metabolically unhealthy obese, cardiometabolic risk

## Abstract

**Introduction:**

Children with obesity in the absence of traditional cardiometabolic risk factors (CRF) have been described as metabolically healthy obese (MHO). Children with MHO phenotype has a favorable metabolic profile with normal glucose metabolism, lipids, and blood pressure compared to children with metabolically unhealthy obese (MUO) phenotype. This study aimed to compare several parameters related to obesity between these two groups and to examine the predictors associated with the MHO phenotype.

**Methods:**

This study included a cross-sectional baseline data of 193 children with obesity (BMI z-score > +2 SD) aged 8-16 years enrolled in MyBFF@school program, a school-based intervention study conducted between January and December 2014. Metabolic status was defined based on the 2018 consensus-based criteria with MHO children had no CRF (HDL-cholesterol > 1.03 mmol/L, triglycerides ≤ 1.7 mmol/L, systolic and diastolic blood pressure ≤ 90^th^ percentile, and fasting plasma glucose ≤ 5.6 mmol/L). Those that did not meet one or more of the above criteria were classified as children with MUO phenotype.

**Results:**

The prevalence of MHO was 30.1% (95% CI 23.7 – 37.1) among schoolchildren with obesity and more common in younger and prepubertal children. Compared to MUO, children with MHO phenotype had significantly lower BMI, lower waist circumference, lower uric acid, higher adiponectin, and higher apolipoprotein A-1 levels (p < 0.01). Multivariate logistic regression showed that adiponectin (OR: 1.33, 95% CI 1.05 – 1.68) and apolipoprotein A-1 (OR: 1.02, 95% CI 1.01 – 1.03) were independent predictors for MHO phenotype in this population.

**Conclusions:**

MHO phenotype was more common in younger and prepubertal children with obesity. Higher serum levels of adiponectin and apolipoprotein A-1 increased the possibility of schoolchildren with obesity to be classified into MHO phenotype.

## Introduction

The worldwide prevalence of childhood obesity continues to rise in many countries (1). Childhood obesity has been linked with many chronic diseases such as type 2 diabetes mellitus, dyslipidemia, hypertension, fatty liver disease (2) and at increased risk of adult mortality (3), hence possesses as one of the most alarming public health concerns. However, studies have shown that not all individuals with obesity exhibit similar degree of obesity-related complications. In recent years, an obese phenotype that has been characterized by the absence of associated cardiometabolic risk factors has been described as metabolically healthy obese (MHO), in contrast to those with metabolically unhealthy obese (MUO) (4). However, the diagnostic criteria of MHO are still under discussion (5), especially in children due to the paucity of the existing studies (6, 7).

Like adult population, studies have suggested that children with obesity can also be characterized as MHO (8, 9) by having a better metabolic profile with normal lipid, glucose metabolism, and blood pressure levels (10). Although children with MHO phenotype does not necessarily means lower morbidity and mortality later in life (7), and can switch to MUO phenotype during puberty (11), defining MHO among children with obesity is crucial in order to elucidate the mechanisms protecting against cardiometabolic risk factors clustering. Therefore, the clear distinction between obese phenotypes could be useful in providing more effective and targeted treatment for children with obesity rather than one-size-fits-all obesity management (12).

Currently, there are no universally accepted criteria to identify children with MHO phenotype despite several attempts have been made using various criteria and cut-off values related to insulin sensitivity and metabolic syndrome components (9, 13–16). In 2018, Damanhoury et al. proposed the first international consensus-based definition of MHO through experts’ consultation and the application of a Delphi process (6). The consensus was achieved to define children with obesity based on body mass index (BMI) for age and gender (BMI z-score) according to the WHO growth chart, and those fulfilling all the cardiometabolic criteria should be classified as MHO. This first step to achieving a universal MHO definition in children is crucial to limit the variability in definitions and to facilitate comparisons across studies (15). Our study contributes to the existing literature by reporting the obesity-related clinical and laboratory parameters among schoolchildren with obesity and to study predictors associated with MHO phenotype using this newly proposed definition.

## Materials and methods

### Study participants

Schoolchildren with obesity (n=193) aged 8-16 years included in this study were from baseline data of My Body is Fit and Fabulous (MyBFF@school) study conducted between January and December 2014 in Malaysia. MyBFF@school study was a school-based lifestyle intervention program specifically designed for overweight and obese schoolchildren to reduce their weight following the 6 months nutritional, physical activity, and psychological modules. Those who were diagnosed with chronic cardiac, renal, hepatic, or endocrine diseases that directly or indirectly related to obesity were excluded from the analysis. Written informed consent and assent were obtained from parents and children respectively, and all tests were performed in accordance with the approved guidelines. The MyBFF@school study was reviewed and approved by the Medical Research and Ethics Committee, Ministry of Health Malaysia. Detailed description of the study methodology has been previously published (17) and the study was also registered with ClinicalTrials.gov (identifier NCT02212873).

### Anthropometric and clinical assessments

Children were asked to fast overnight for at least 8 hours prior to study visit. All anthropometric measurements were performed by trained personnel and medical assessments were performed by qualified pediatricians. Height was measured while standing without shoes to the nearest 0.1 cm using calibrated stadiometer (Seca 217, Germany). Body composition such as weight, fat mass and skeletal muscle mass was measured in light clothing without shoes and socks to the nearest 0.1 kg using a pre-calibrated bioelectrical impedance analyzer (InBody 720, Korea). BMI was calculated as weight in kilograms (kg) divided by the square of height in meters (m^2^). Waist circumference was measured twice to the nearest 0.1 cm over the skin midpoint between the tenth rib and the iliac crest at the end of normal exhalation, using an inelastic measuring tape (Seca 201, Germany) with the children standing still on both feet with arms hanging freely. Systolic (SBP) and diastolic blood pressure (DBP) were measured twice on the right arm using a mercury sphygmomanometer (Accoson, UK) after 5 minutes of rest in a seated position with the arm supported at the heart level. Self-assessment of pubertal status was performed using pictorial Tanner staging scale (18, 19) and the children were also examined by the pediatricians for the presence of acanthosis nigricans over the neck (20).

### Biochemical measurements

Venipuncture was performed by experienced nurses and medical doctors. Venous blood samples were kept cold and processed within 2 hours upon sample collection at the Institute for Medical Research central laboratory, and aliquots of serum/plasma were kept at -20°C for short-term storage or -80°C for long-term storage prior to analysis. Fasting plasma glucose (FPG), total cholesterol (TC), triglycerides (TG), high-density lipoprotein-cholesterol (HDL-cholesterol), low-density lipoprotein-cholesterol (LDL-cholesterol), apolipoprotein A-1 (apo A-1), apolipoprotein B (apo B), high-sensitivity C-reactive protein (hsCRP), adiponectin, uric acid (UA), alanine transaminase (ALT), aspartate transaminase (AST), gamma-glutamyl transferase (GGT) and serum creatinine were measured using an automated analyzer (Dirui CS-400, China) with reagents purchased from Randox Laboratories (Antrim, UK). HbA1c level was determined by cationic exchanged high-performance liquid chromatography (Adams A1c HA-8160, Arkray Inc., Japan) following the National Glycohemoglobin Standardization Program guidelines. Fasting insulin concentration was measured using an automated enzyme immunoassay analyzer (TOSOH AIA-360, Japan). Leptin and interleukin-6 (IL-6) were measured by an enzyme-linked immunosorbent assay (ELISA) commercial kit with sensitivity of 0.7 ng/mL (IBL International, Germany) and sensitivity of 0.4 pg/mL (R&D Systems, USA) respectively. Both ELISA assays have intra-and inter-assay coefficient of variation less than 10%.

### Definitions of measures

Obesity was defined as BMI z-score > +2 standard deviation (SD) according to the WHO growth chart. All children in this study were divided into those with MHO and MUO according to the consensus-based definition proposed by Damanhoury et al. (6). Children were classified as MHO if they met all the following criteria: HDL-C > 1.03 mmol/L, TG ≤ 1.7 mmol/L, SBP and DBP ≤ 90^th^ percentile, and FPG ≤ 5.6 mmol/L. Since no consensus was achieved regarding a measure of glycemia, FPG ≤ 5.6 mmol/L was chosen because it was most used in previous studies of MHO in children (10). Children with obesity that did not meet one or more of the above criteria were classified as MUO. Prepubertal was classified as Tanner stage 1 external genitalia development for boys and breast development for girls, while stage 2 and above were defined as pubertal. Abdominal obesity was defined as waist circumference ≥ 90^th^ percentile of the Malaysian children (21). Homeostasis Model Assessment of Insulin Resistance (HOMA-IR) index was calculated as previously described (22), with insulin resistance defined as HOMA-IR index ≥ 2.6 and ≥ 4.0 for prepubertal and pubertal children respectively (23, 24). AST : ALT ratio less than 1 was considered as a high risk for non-alcoholic fatty liver disease (NAFLD) (25). The clinical practice guidelines for screening and management of high blood pressure in children and adolescents (26) was used to calculate blood pressure percentile. Estimated glomerular filtration rate (eGFR) was calculated using modified Schwartz formula (27) with abnormal eGFR defined as < 75 mL/min/1.73m^2^ (28).

### Statistical analysis

The normality test for continuous data was determined using the Kolmogorov-Smirnov test. Continuous variables were presented as mean (standard deviation) for normally distributed and median (25^th^ percentile, 75^th^ percentile) for non-normally distributed variables, and the differences between obese phenotypes were compared using independent t-test and Mann-Whitney test respectively. Categorical variables were presented as frequency and proportion and comparisons between groups were made using the chi-square test. Multivariate logistic regression was performed and measured by calculating the odds ratio (OR) and 95% confidence interval (95% CI) to identify the predictors associated with MHO phenotype using Hosmer Lemeshow Model building strategy. All analyses were performed using IBM SPSS 26.0 and statistical significance was set at 2-sided *P* < 0.05.

## Results

**Figure 1:**
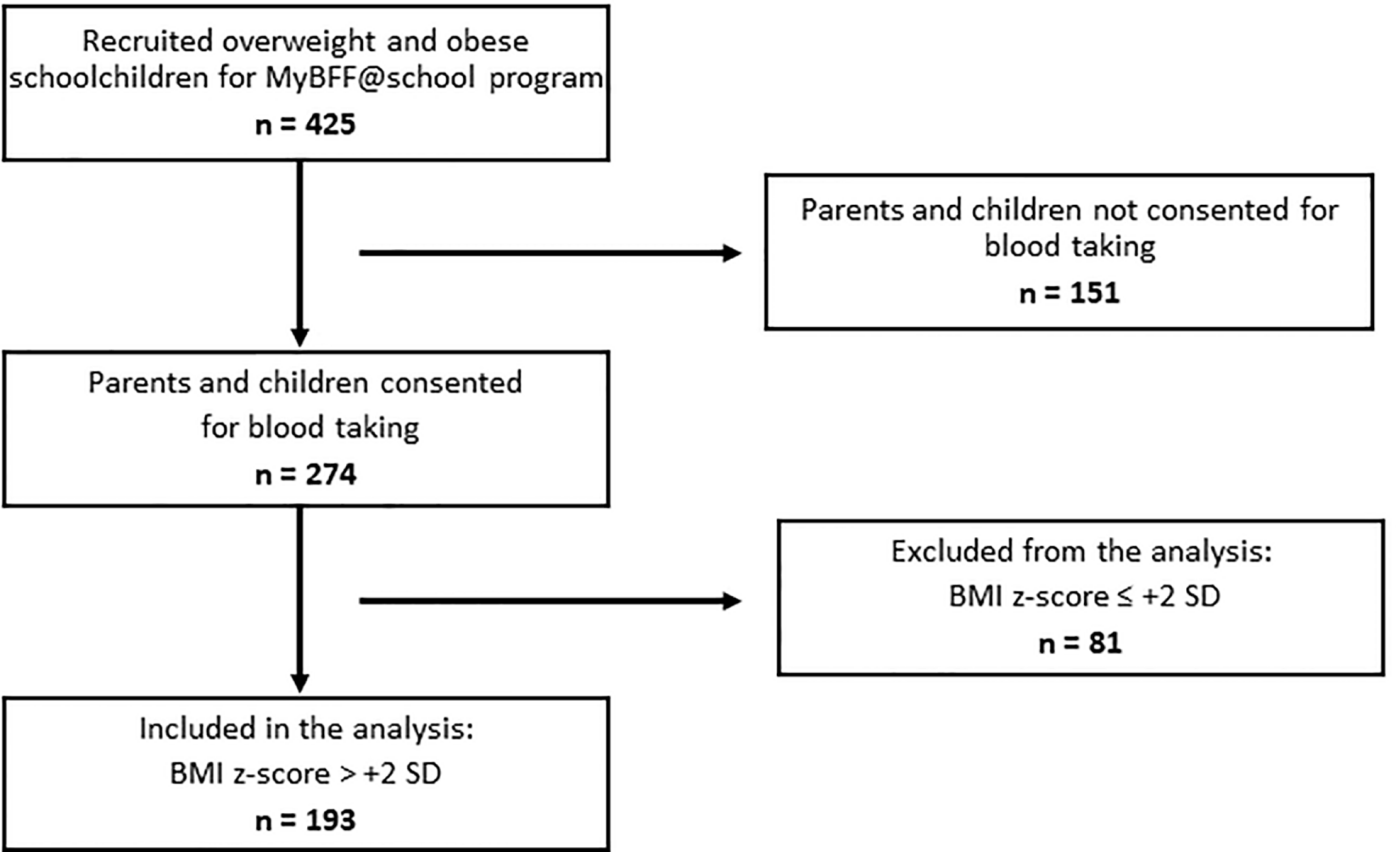
Flow chart for blood selection for MHO and MUO analysis.

A total of 425 overweight and obese Malay schoolchildren participated in the MyBFF@school study, of which 274 (65%) consented for blood taking. Out of these, baseline data of 193 children with obesity with BMI z-score > +2 SD were analyzed for obese phenotypes and obesity related clinical and laboratory parameters (**Figure 1**). The prevalence of MHO among schoolchildren with obesity was 30.1% (95% CI 23.7 – 37.1%). The values of parameters used to characterize children as either MHO or MUO were statistically significant between the two groups except for BMI z-score median [2.64 (2.46, 3.01) vs 2.85 (2.46, 3.17), p-value = 0.16] (**Table 1**). Among children with MUO phenotype, 88 (65.2%) presented only one risk factor, 38 (28.1%) 2 risk factors, 8 (5.9%) 3 risk factors and only 1 child (0.7%) had all four risk factors. High blood pressure (SBP or DBP > 90^th^ percentile) was the most represented risk factor (51.9%) among MUO children and was significantly higher in boys than in girls (60.0% vs 41.7%, p-value = 0.03). Instead, elevated triglycerides (TG > 1.7 mmol/L) was the least observed risk factor among children with MUO phenotype (**Table 2**).

**Table 1:** Comparison of parameters used as criteria to define metabolically healthy obese (MHO) and metabolically unhealthy obese (MUO) phenotypes.

	MHO	MUO	p-value	Total n = 193
	n = 58	n = 135	
BMI z-score, median (q25, q75)	2.64 (2.46, 3.01)	2.85 (2.46, 3.17)	0.16	2.77 (2.46, 3.11)
HDL-cholesterol (mmol/L), mean (SD)	1.25 (0.16)	1.09 (0.21)	< 0.001	1.14 (0.21)
Triglycerides (mmol/L), mean (SD)	0.96 (0.31)	1.19 (0.50)	< 0.001	1.12 (0.46)
Systolic BP (mm Hg), mean (SD)	104 (9)	112 (11)	< 0.001	110 (11)
Diastolic BP (mm Hg), mean (SD)	64 (7)	73 (9)	< 0.001	70 (10)
Fasting plasma glucose (mmol/L), mean (SD)	5.13 (0.36)	5.37 (0.48)	< 0.001	5.30 (0.46)

Data are expressed as mean (standard deviation) for normally distributed data or median (25^th^ percentile, 75^th^ percentile) for non-normally distributed data. Independent t-test (normally distributed data) or Mann-Whitney test (non-normally distributed data) were used to compare differences between MHO and MUO children. BMI, body mass index; HDL-cholesterol, high-density lipoprotein-cholesterol; BP, blood pressure; MHO, metabolically healthy obese; MUO, metabolically unhealthy obese; SD, standard deviation; q25, 25^th^ percentile; q75, 75^th^ percentile.

**Table 2:** Distribution of cardiometabolic risk factors among MUO children by gender.

	Girls	Boys	p-value	Total n = 135
	n = 60	n = 75	
HDL-cholesterol ≤ 1.03 mmol/L	30 (50.0%)	36 (48.0%)	0.82	66 (48.9%)
Triglycerides > 1.7 mmol/L	11 (18.3%)	10 (13.3%)	0.43	21 (15.6%)
Systolic & diastolic BP > 90^th^ percentile	25 (41.7%)	45 (60.0%)	0.03	70 (51.9%)
Fasting plasma glucose > 5.6 mmol/L	12 (20.0%)	23 (30.7%)	0.16	35 (25.9%)

Data presented as frequency and proportion (%). Chi-square test was used to compare the cardiometabolic risk distribution among MUO children based on gender. HDL-cholesterol, high-density lipoprotein-cholesterol; BP, blood pressure.

Obesity-related clinical and laboratory parameters were presented in **Table 3**. Children with MHO phenotype was significantly younger [11.9 (2.2) vs 12.6 (2.1) years, p-value = 0.03)], had significantly lower BMI [28.4 (3.3) vs 30.0 (3.9) kg/m2, p-value < 0.01] and had significantly lower waist circumference [86.6 (8.5) vs 91.3 (9.8), p-value < 0.01)] as compared to children with MUO phenotype. Moreover, MHO phenotype was more common among prepubertal children with obesity (p-value = 0.02). The presence of acanthosis nigricans (physical marker of insulin resistance) was more common among MUO than in MHO children (p-value = 0.03). Serum apolipoprotein A-1 and adiponectin were significantly higher (p-value < 0.01) in MHO as compared to MUO children with values of 189.7 (36.2) vs 172.3 (34.5) mmol/L and 7.1 (2.4) vs 5.5 (2.1) µg/mL respectively. In addition, serum uric acid was significantly lower in MHO as compared to MUO children [364.7 (65.1) vs 389.5 (65.8) µmol/L, p-value = 0.04)].

**Table 3:** Obesity-related clinical and laboratory parameters examined in MHO and MUO children.

	MHO	MUO	p-value	Total n = 193
	n = 58	n = 135	
Age (years), mean (SD)	11.9 (2.2)	12.6 (2.1)	0.03	12.4 (2.1)
Gender, n (%)				
Girls	32 (55.2)	60 (44.4)	0.17	92 (47.7)
Boys	26 (44.8)	75 (55.6)		101 (52.3)
Puberty status, n (%)				
Prepubertal	28 (49.1)	41 (30.8)	0.02	69 (36.3)
Pubertal	29 (50.9)	92 (69.2)		121 (63.7)
Adiposity measures				
BMI (kg/m^2^), mean (SD)	28.4 (3.3)	30.0 (3.9)	< 0.01	29.5 (3.8)
Waist circumference (cm), mean (SD)	86.6 (8.5)	91.3 (9.8)	< 0.01	89.9 (9.6)
Abdominal obesity (WC > 90^th^ percentile), n (%)	51 (98.1)	126 (99.2)	0.51	177 (98.9)
Skeletal muscle mass, mean (SD)	19.5 (5.2)	21.8 (5.4)	< 0.01	21.1 (5.4)
Body fat mass, mean (SD)	26.9 (8.0)	29.8 (7.9)	0.02	28.9 (8.0)
Body fat percentage, mean (SD)	42.3 (5.1)	42.3 (4.9)	0.97	42.3 (4.9)
Glycaemic measures				
Fasting insulin (µU/mL), median (q25, q75)	14.8 (10.2, 23.6)	17.0 (11.0, 24.7)	0.24	16.7 (10.7, 24.6)
HOMA-IR, median (q25, q75)	3.4 (2.3, 5.3)	4.0 (2.7, 5.6)	0.22	3.9 (2.4, 5.6)
Insulin Resistance, n (%)	28 (48.3)	78 (57.8)	0.16	106 (54.9)
HbA1c (%), mean (SD)	5.2 (0.3)	5.2 (0.3)	0.43	5.2 (0.3)
Acanthosis nigricans, n (%)	28 (49.1)	87 (65.4)	0.03	115 (60.5)
Lipid metabolism				
Total cholesterol (mmol/L), mean (SD)	4.67 (0.57)	4.45 (0.86)	0.05	4.52 (0.79)
LDL-cholesterol (mmol/L), mean (SD)	3.20 (0.73)	3.18 (0.87)	0.92	3.19 (0.83)
Apolipoprotein A-1 (mmol/L), mean (SD)	189.7 (36.2)	172.3 (34.5)	< 0.01	177.2 (35.7)
Apolipoprotein B (mmol/L), mean (SD)	90.4 (20.9)	96.8 (26.4)	0.35	94.9 (25.1)
Pro-inflammatory markers				
hsCRP (mg/L), median (q25, q75)	1.6 (0.6, 4.3)	2.8 (0.7, 5.0)	0.44	2.7 (0.7, 4.7)
Uric acid (µmol/L), mean (SD)	364.7 (65.1)	389.5 (65.8)	0.04	382.3 (66.3)
Adipokines				
Adiponectin (µg/mL), mean (SD)	7.1 (2.4)	5.5 (2.1)	< 0.01	5.9 (2.3)
Leptin (ng/mL), mean (SD)	24.8 (11.1)	25.7 (11.5)	0.73	25.5 (11.3)
Interleukin-6 (pg/mL), median (q25, q75)	1.7 (1.2, 3.1)	2.3 (1.6, 3.4)	0.09	2.1 (1.5, 3.3)
Liver function marker				
ALT (U/L), mean (SD)	13.4 (7.4)	15.4 (8.5)	0.12	14.8 (8.2)
AST (U/L), mean (SD)	21.3 (7.7)	20.9 (7.6)	0.81	21.1 (7.6)
AST : ALT ratio, mean (SD)	1.65 (0.66)	1.49 (0.62)	0.12	1.54 (0.63)
NAFLD risk (AST: ALT < 1), n (%)	8 (14.8)	23 (18.3)	0.57	31 (17.2)
GGT (U/L), mean (SD)	22.2 (10.9)	23.6 (9.8)	0.37	23.2 (10.2)
Kidney function				
Serum creatinine (µmol/L), mean (SD)	75.4 (12.7)	77.2 (10.9)	0.42	76.7 (11.5)
eGFR (mL/min/1.73m^2^), mean (SD)	72.2 (10.8)	71.8 (10.5)	0.66	71.6 (9.9)
Risk of CKD (eGFR < 75 mL/min/1.73m^2^), n (%)	26 (70.3)	70 (72.9)	0.76	96 (72.2)

Data are expressed as mean (standard deviation) for normally distributed data, median (25^th^ percentile, 75^th^ percentile) for non-normally distributed data or proportion (%) for categorical data. Independent t-test (normally distributed data), Mann-Whitney test (non-normally distributed data) or chi-square test (categorical data) were used to compare differences between MHO and MUO children. MHO, metabolically healthy obese; MUO, metabolically unhealthy obese; BMI, body mass index; WC, waist circumference; HOMA-IR, homeostasis model assessment of insulin resistance; LDL-cholesterol, low-density lipoprotein-cholesterol; hsCRP, high-sensitivity C-reactive protein; ALT, alanine transaminase; AST, aspartate transaminase; NAFLD, non-alcoholic fatty liver disease; GGT, gamma-glutamyl transferase; eGFR, estimated glomerular filtration rate; CKD, chronic kidney disease; SD, standard deviation; q25, 25^th^ percentile; q75, 75^th^ percentile

**Figure 2:**
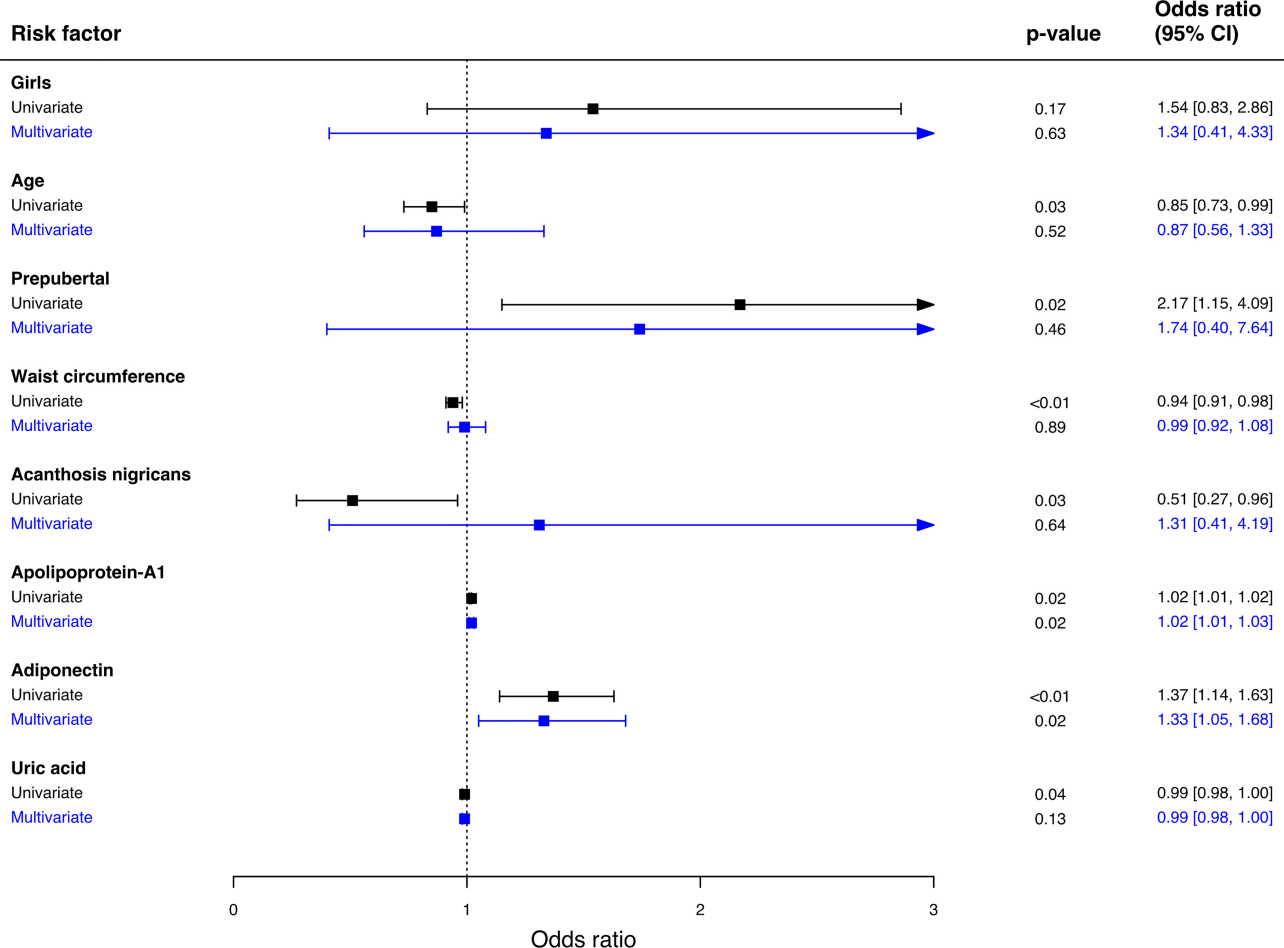
Predictors associated with MHO phenotype. Multivariate logistic regression was used to analyze the association between variables and MHO phenotype. Hosmer Lemeshow Test (chi-square = 5.32, p-value = 0.72), classification table, overall correctly classified percentage = 80.4. 95% CI; 95% confidence interval.

Multivariate logistic regression was used to examine the predictors associated with MHO phenotype (**Figure 2**). Adiponectin and apolipoprotein A-1 are independent predictors for MHO phenotype in schoolchildren with obesity. The probability of being classified as MHO increased by 33% for each increment of 1µg/mL serum adiponectin [(OR: 1.33, 95% CI 1.05 – 1.68), p-value = 0.02], and by 2% for each increment of 1mmol/L serum apolipoprotein A-1 [(OR: 1.02, 95% CI 1.01 – 1.03), p-value = 0.02].

## Discussion

Using the recently proposed consensus-based definition (6), our study demonstrated that the prevalence of MHO was 30.1% (95% CI 23.7 – 37.1) among schoolchildren with obesity in the community, and it was more common among younger and prepubertal children. Compared to MUO, MHO children had significantly lower BMI, lower waist circumference, lower serum uric acid, higher serum adiponectin and higher serum apolipoprotein A-1 levels. Multivariate logistic regression showed that adiponectin and apolipoprotein A-1 were the independent predictors for MHO phenotype in this population.

Similar to other studies, our findings showed that MHO is more common in younger (9, 29–31) and prepubertal (11, 13) children with obesity. These observations may be explained by the physiological and pathological changes children undergo during puberty, which includes a decrease in insulin sensitivity (32). A longitudinal study reported that entering from pre-to mid-puberty caused an increased in insulin resistance and likelihood of switching from MHO to the MUO phenotype, whereas as children progressed from mid-to late puberty, insulin resistance decreased and the likelihood for crossing over from MUO to MHO increased (11). Furthermore, MHO phenotype was found to be more common in girls than in boys (13, 30, 33), and this may be attributed by differences in hormone levels, lifestyle and body fat distribution (34, 35), although the mechanisms are not fully understood. However, we did not see this discrepancy between girls and boys in our study.

Although no consensus was achieved for glycemic measures in the definition by Damanhoury et al., fasting plasma glucose with cut-off of ≤ 5.6 mmol/L was widely used in previous studies of MHO in children (6). In this study, children with MUO phenotype were highly presented with acanthosis nigricans than in children with MHO phenotype despite no significant difference was observed in fasting insulin, HOMA-IR and HbA1c between groups. Evidence indicates that acanthosis nigricans is a marker of glucose metabolism derangement and was associated with insulin resistance in children with obesity (36). Although the hyperinsulinemic-euglycemic clamp is considered the gold standard for the identification of insulin resistance, due to its complexity and invasiveness, the most widespread method for assessing insulin resistance among children is through the calculation of HOMA-IR. Studies have found that children with MHO phenotype had lower HOMA-IR index as compared to children with MUO phenotype (13, 29, 31, 37, 38), however we did not observe this trend and our finding agrees with other more recent studies (39, 40). A study found that both children with MHO and MUO phenotype had significantly higher HOMA-IR when compared to normal weight peers indicating that a state of insulin resistance was already present (40). It is also noteworthy to mention that half of our study population showed insulin resistance. This may suggest that rather than obesity per se, possible differences in the genetic predisposition of each child to develop insulin resistance could also play a role, however, this is beyond the scope of this paper.

Adiposity measures such as BMI and waist circumference have been reported to be independent predictors of MHO phenotype (9, 41, 42). Although our study found that BMI and waist circumference were significantly lower among children with MHO phenotype when compared to MUO, these two adiposity measures were not predictors for MHO phenotype in this studied population. Our findings concur with study by Ooi et al. (29) and this may suggest that there may be other underlying factors that contribute to the MHO phenotype, albeit obesity is a risk factor for metabolic morbidities (43). In addition, higher BMI observed among MUO in our study may be due to higher skeletal muscle mass and fat mass. Indeed, BMI has been reported as a poor predictor of adiposity in children with obesity due to the inability to distinguish between body fat mass and lean tissue mass (44).

Growing evidence suggests that obesity causes low-grade chronic inflammation which adipose tissue inflammation is thought to be involved in the pathogenesis of insulin resistance and cardiovascular complications (45). Adults with MHO phenotype were shown to have better pro-inflammatory markers such as hsCRP and IL-6 compared to MUO phenotype (46), however the association in children remains controversial. We did not observe any significant differences in serum hsCRP and IL-6 between MHO and MUO children, and our findings concur with Serbis et al. (40). Nevertheless, they found that pro-inflammatory markers were significantly elevated among children with obesity when compared to normal weight children, suggesting that obesity is associated with subclinical inflammation even in children with MHO phenotype (40). A more recent study found that hsCRP but not IL-6 was a predictor for MHO children (47). On the other hand, adiponectin, an anti-inflammatory protein was found to be significantly higher among MHO than in MUO children in our study. Multivariate logistic regression showed that adiponectin was an independent predictor for MHO phenotype in this population, and our finding agrees with a study by Fu et al. (48). Higher serum levels of adiponectin have been reported to have a protective effect against cardiovascular risks and was associated with preserved insulin sensitivity in children with obesity (49, 50).

This study found a significantly lower serum uric acid among children with MHO phenotype as compared to children with MUO phenotype. Hyperuricemia was linked to increased risk of developing metabolic syndrome, type 2 diabetes, and cardiovascular diseases in children with obesity (51). Studies have reported that serum uric acid was an independent predictor for MHO phenotype in children with obesity (37, 42), however our regression model could not replicate similar findings. Concerning apolipoprotein A-1, children with MHO phenotype showed significantly higher concentration than children with MUO phenotype in this study. Low concentration of apolipoprotein A-1 is linked to endothelial dysfunction in children with obesity (52). In addition, apolipoprotein A-1 was found to be an independent predictor for MHO phenotype in our studied population, however, this association must be interpreted cautiously as the odd ratio and its 95% confidence interval reaching null value.

Non-alcoholic fatty liver disease (NAFLD) has been linked with obesity, and ALT is the best screening tool to detect NAFLD in children (53). A study found that obese children with metabolic syndrome are more likely to have advanced liver fibrosis compared to those without metabolic syndrome (54). However, none of the liver function markers showed any differences between MHO and MUO children in this study, implying that not only MUO but also those with MHO phenotype are at risk of developing liver dysfunction and possibly NAFLD. Indeed, our studied population showed that 17.2% are at risk of developing NAFLD.

Studies have shown that adults with MHO phenotype are at increased risk of developing chronic kidney disease (CKD) when compared to normal weight counterparts (55, 56). Even though the evidence is not as robust in children, there are several studies that reported risk of developing CKD with obesity (57, 58). Study by Arora et al. found no significant difference in the serum creatinine and eGFR between MHO and MUO children (59), and this is consistent with our findings. However, it should be noted that 72.2% of these schoolchildren with obesity were at increased risk of developing CKD (eGFR < 75 mL/min/1.73m2), thus proves that MHO phenotype is not a benign condition and is associated with long-term development of CKD.

One of the advantages of using the consensus-based definition is the fact that most of the cut-off values were adapted from the definition of metabolic syndrome in children provided by the International Diabetes Federation (IDF) (60), and therefore has been widely used by pediatricians and researchers dealing with childhood obesity. These cut-off values have been shown reliable in children with obesity (6, 15, 29) and therefore should facilitate comparison with future studies to avoid further diversification in defining MHO among children with obesity.

Despite the notable findings in this study, there are several limitations that need to be addressed. Firstly, this study has a relatively small sample size from an epidemiological point of view and employed a cross-sectional design, hence unable to establish the causal relationship between predictors and MHO phenotype. Secondly, it could be speculated that there may be differences in fat distribution between obese phenotypes since reference methods of adiposity estimation were not used, and visceral fat accumulation has been reported to be directly linked with metabolic health (61). Lastly, information on modifiable factors such as diet and lifestyle habits were not available in this study. One of the strengths of this study was that the study population was obtained from community screening. Unlike other studies, most were done at hospital setting (11, 13, 29, 31, 37–40) where the children were already referred to the obesity clinic by their pediatricians. In addition, we measured extended obesity-related biochemical parameters to corroborate that MHO is not a totally benign condition after all.

In conclusion, our study found that one third of children with obesity had MHO phenotype and had better adiponectin and apolipoprotein A-1 levels. However, no differences were observed for other obesity-related blood parameters. Although the definition by Damanhoury et al. (6) can be potentially useful in distinguishing between the MHO and MUO phenotypes in children, this may lead to underestimating the number of children at risk for other obesity-related diseases such as fatty liver and CKD, even in the absence of the traditional cardiometabolic risks. Hence, a more comprehensive and stringent definition is needed, and until then, children with MHO phenotype should be treated similar to all children with obesity.

## Data availability statement

The raw data supporting the conclusions of this article will be made available by the authors, without undue reservation.

## Ethics statement

The studies involving human participants were reviewed and approved by Medical Research and Ethics Committee, Ministry of Health Malaysia. Written informed consent to participate in this study was provided by the participants’ legal guardian/next of kin.

## Author contributions

WNWM was the principal researcher and was responsible for the overall conception and design of the project, seeking funding, and coordinating with schools for data collection. RMWMZ and AKNZI were responsible for the logistics, data collection, laboratory analysis of samples, data management and statistical analysis. MYJ, FMZ, and JYHH were responsible for conceptualizing the clinical data collection and conducted the clinical examinations on the study participants. AY was responsible for sample size calculation and statistical analysis. AHM was responsible for the conception and design of the project. All authors contributed to the article and approved the submitted version.

## Funding

This study was fully funded by Ministry of Health Malaysia (grant number: NMRR-13-439-16563).

## Acknowledgments

The authors would like to thank the Director General of Health Malaysia for his permission to publish this article, Dr Othman Warijo, Dr Husni Hussain, and Ministry of Education for their active support. We also would like to express our gratitude to children and parents for agreeing to participate in the MyBFF@school program.

## Conflict of interest

The authors declare that the research was conducted in the absence of any commercial or financial relationships that could be construed as a potential conflict of interest.

## Publisher’s note

All claims expressed in this article are solely those of the authors and do not necessarily represent those of their affiliated organizations, or those of the publisher, the editors and the reviewers. Any product that may be evaluated in this article, or claim that may be made by its manufacturer, is not guaranteed or endorsed by the publisher.
